# Contribution of Telomere Length to Systemic Sclerosis Onset: A Mendelian Randomization Study

**DOI:** 10.3390/ijms242115589

**Published:** 2023-10-25

**Authors:** Inmaculada Rodriguez-Martin, Gonzalo Villanueva-Martin, Alfredo Guillen-Del-Castillo, Norberto Ortego-Centeno, José L. Callejas, Carmen P. Simeón-Aznar, Javier Martin, Marialbert Acosta-Herrera

**Affiliations:** 1 Institute of Parasitology and Biomedicine López-Neyra, CSIC, 18016 Granada, Spain; 2 Department of Internal Medicine, Hospital Universitari Vall d’Hebron, 08035 Barcelona, Spain; 3 Systemic Autoimmune Disease Unit, Hospital Clínico San Cecilio, Instituto de Investigación Biosanitaria Ibs. GRANADA, 18012 Granada, Spain; 4 Department of Medicine, University of Granada, 18016 Granada, Spain

**Keywords:** systemic sclerosis, telomere length, mendelian randomization

## Abstract

Although previous studies have suggested a relationship between telomere shortening and systemic sclerosis (SSc), the association between these two traits remains poorly understood. The objective of this study was to assess the causal relationship between telomere length in leukocytes (LTL) and SSc using the two-sample Mendelian randomization approach, with the genome-wide association study data for both LTL and SSc. The results of inverse-variance weighted regression (OR = 0.716 [95% CI 0.528–0.970], *p* = 0.031) and the Mendelian randomization pleiotropy residual sum and outlier method (OR = 0.716 [95% CI 0.563–0.911], *p* = 0.035) indicate an association between telomere length and SSc. Specifically, longer genetically predicted LTL is associated with a reduced risk of SSc. Sensitivity tests highlight the significant roles of the variants rs10936599 and rs2736100 annotated to the *TERC* and *TERT* genes, respectively. Our findings suggest an influence of telomere length in leukocytes on the development of SSc.

## 1. Introduction

Systemic sclerosis (SSc) is an immune-mediated inflammatory disease (IMID) characterized by vascular damage, chronic inflammation and fibrotic involvement of connective tissues [1]. SSc patients can be classified as limited cutaneous SSc (lcSSc) or diffuse cutaneous SSc (dcSSc) based on the extension of skin fibrosis [2]. Similarly to other IMIDs, SSc is a complex disease involving interplay of genetic, epigenetic and environmental factors [2].

Significant progress has been made in the genetic understanding of SSc in recent years through extensive genome-wide association studies (GWAS) and Immunochip studies [3,4,5]. The majority of the robustly replicated SSc susceptibility loci are involved in innate or adaptive immune system [6]. These studies, coupled with comprehensive expression analyses, have underscored the involvement of leukocytes in SSc pathogenesis [3,4,5,6,7,8,9].

Telomeres are nucleoprotein structures located at chromosome ends that are implicated in the preservation of genome integrity and stability [10]. These structures naturally undergo telomere shortening (TS), a process linked to inflammation and cellular senescence, both of which are implicated in the pathogenesis of SSc [2,11,12,13,14]. Of note, previous studies evaluating telomere length in leukocytes (LTL) in SSc have been undertaken; however, the findings have been inconsistent, and their significance regarding SSc pathogenesis remains unclear [15,16,17,18,19].

Mendelian randomization (MR) studies allow for exploring the potential causal relationship between a risk factor and a disease [20]. These studies employ single-nucleotide polymorphisms (SNPs) as instrumental variables (IVs) and are based on the assumption that SNPs are associated with the risk factor but not with the disease or a confounding factor [20]. In recent years, MR studies have been conducted to overcome the limitations of observational studies and to provide insights into the causal relationship between several IMIDs and multiple exposures [21].

In the present study, we aimed to investigate the causal relationship between LTL and SSc through an MR study.

## 2. Results

Seven IVs were used in our study based on their association with LTL [22]. This information and their respective effect estimators for the SSc and SSc clinical subtypes are shown in Table 1 and Table S1, respectively. The selected IVs were strong enough to avoid weak bias, as indicated by the F-statistic value of 67.03 [23].

Since none of the heterogeneity tests were significant, the inverse-variance weighted (IVW) fixed-effects (FE) method was used for the three datasets analyzed (Table 2 and Table 3). In addition, MR-Egger showed no evidence of horizontal pleiotropy in any of the datasets, and the MR pleiotropy residual sum and outlier (MR-PRESSO) method detected no outliers (Table 2 and Table 3).

Our results for IVW-FE show an association between genetically predicted longer LTL and a reduced risk of SSc (OR = 0.716 [95% CI 0.528–0.970], *p* = 0.031). In addition, three other MR methods, maximum likelihood, weighted median and MR-PRESSO, showed the consistency of our results, with a significant association and the same direction of effect (Table 2). Interestingly, even when weighted mode regression did not reach statistical significance, the estimate was in the same direction as the other methods. MR-Egger regression results were not statistically significant (OR = 0.397 [95% CI 0.094–1.670], *p* = 0.263). Table 2 summarizes the MR results for LTL and SSc, indicating a negative relation between genetically predicted telomere length and SSc. Regarding leave-one-out (LOO) sensitivity analysis, we observed that individually removing two SNPs (rs10936599 and rs2736100) from the analysis resulted in the loss of the significance, maintaining the direction of the effect (*p* = 0.241 and *p* = 0.102, respectively; Table S2).

Lastly, we investigated the specific associations for LTL with the two main clinical subtypes of the disease. Our results show a significant association between genetically predicted longer LTL and a decreased risk of lcSSc for IVW-FE with an OR of 0.669 [95% CI 0.468–0.956] and a *p*-value of 0.027. In addition, the maximum likelihood, weighted median and MR-PRESSO methods were also statistically significant (Table 3). In contrast, the results in dcSSc showed no statistically significant association with LTL for any of the MR methods applied (IVW-FE: OR = 0.771 [95% CI 0.481–1.237], *p* = 0.281; Table 3). Noteworthy, LOO sensitivity analysis results for lcSSc revealed the loss of significance for the same two SNPs as observed for the complete SSc dataset, rs10936599 *p* = 0.234 and rs2736100 *p* = 0.081 (Table S3).

## 3. Discussion

In the current study, we investigated the possible causal relationship of LTL with the risk of SSc, and its main clinical subtypes, using the MR methodology and the largest SSc GWAS in Europeans [3]. Our primary findings show a significant association between genetically predicted longer LTL and a reduced risk of SSc, shedding light on the direction of influence between these two traits.

Previous observational studies in LTL in SSc reached conflicting results [15,16,17]. However, the largest and most recent study described a higher proportion of patients with SSc with shorter telomeres compared to controls [15]. Consistent with this observation, our data indicate that genetically predicted longer LTLs are associated with a reduced risk of SSc (IVW-FE: OR = 0.716 [95% CI 0.528–0.970], *p* = 0.031; Table 2). Furthermore, owing to the methodology applied, our study provides evidence that LTL influences the risk of developing SSc rather than the disease progression affecting LTL. These results align with the data reported in a MR study for LTL performed for an IMID primarily affecting the skin, psoriasis [24], and another connective tissue disease, rheumatoid arthritis [25]. However, our results significantly contrast with the results of a similar study in systemic lupus erythematosus [26]. These discrepancies may be explained by the high complexity of connective tissue disorders.

Regarding the relationship of LTL with the main clinical subtypes, we observed a significant association between LTL and lcSSc, whereas the analysis for dcSSc did not reach statistical significance (Table 3). This observation may be attributed to variations in statistical power between clinical subtypes, as shown in Table S4. Several studies have analyzed LTL in lcSSc and dcSSc, yielding conflicting results [15,18,19] that may be related to the different sample sizes, normal telomere length (TL) reference or methods used to measure TL. These inconsistencies underscore the necessity for further and larger studies to understand LTL in lcSSc and dcSSc.

In examining the sensitivity analyses, we found that two SNPs (rs10936599 and rs2736100) were the primary contributors to the obtained results. Interestingly, these SNPs and their corresponding annotated genes, *TERC* and *TERT*, were associated with idiopathic forms of interstitial lung disease [27,28]. Moreover, it is worth nothing that telomere shortening is an established risk factor for idiopathic pulmonary fibrosis [14,24] and has also been associated with different pulmonary features in SSc patients [15,18]. Taking the above into consideration, the contribution of LTL to SSc seems to be especially relevant in the severe pulmonary affection of the disease. Unfortunately, the clinical data regarding pulmonary affection in our large GWAS cohort are limited, and we could not evaluate this in the present study.

Our results underscore the potential involvement of LTL in SSc development. However, the mechanism by which LTL affects SSc remains unknown. In this context, we hypothesize some potential pathways. First, short TL could contribute to the dysregulation of the immune system by affecting T-cell numbers and receptor diversity [28]. Notably, CD28-negative T cells that have a proinflammatory profile with alternative receptors, cytolytic properties and shorter TL [13] have been reported to be increased in the blood and skin of SSc patients [29]. Additionally, several studies have related short TL and telomere dysfunction with increased proinflammatory cytokines [30,31,32], which could contribute to the immune imbalance of the disease. Furthermore, the presence of autoantibodies against a telomere-related protein (TERF1) in some SSc patients and its association with shorter LTL [33] highlight the interplay between telomeres and immune response.

## 4. Materials and Methods

**Figure 1:**
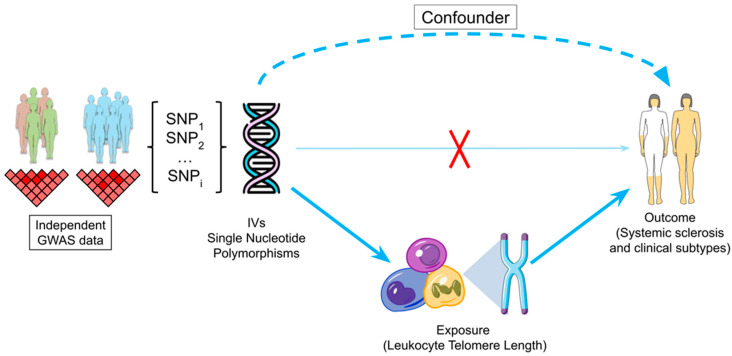
Framework of the 2SMR study for leukocyte telomere length and systemic sclerosis. The 2SMR approach utilizes data from two independent GWAS and employs SNPs as instrumental variables. The selected SNPs are associated with the exposure, influence the outcome only through the exposure and should not be associated with any confounder. Parts of the figure were drawn by using and modifying pictures from Servier Medical Art. Servier Medical Art by Servier is licensed under a Creative Commons Attribution 3.0 Unported License (https://creativecommons.org/licenses/by/3.0/), accessed on 26 September 2023. 2SMR: two-sample Mendelian randomization; GWAS: genome-wide association studies; IV: instrumental variable; SNP: single-nucleotide polymorphism.

To determine the possible causal relationship between LTL and SSc and its main clinical subtypes, lcSSc and dcSSc, we carried out a two-sample MR (2SMR) study (Figure 1). This approach allows the use of two non-overlapping datasets, one for the exposure and another for the outcome, to determine the causal relationship between them [34].

### 4.1. Genetic Data Sources and IV Selection

Genetic association data for seven non-palindromic independent SNPs, associated at genome-wide significance level (*p* < 5 × 10^−8^) with LTL, were obtained from a GWAS meta-analysis comprising 37,684 European-descent individuals [22]. Summary statistics from the largest SSc GWAS in Europeans (9095 patients with SSc and 17,584 controls) [3] were used to extract the association estimates for the outcome. A summary of these SNPs as IVs and their size effect on both LTL and SSc can be found in Table 1. In addition, association data specific for lcSSc and dcSSc were also used (Table S1). The sample size of the cohort and the clinical subtypes are shown in Table S5.

To ensure the adequacy of the IVs, Phenoscanner [35,36] and LDtrait [37,38] were used to verify the absence of a direct association of the IVs with the outcome. The strength of the selected IVs was evaluated using the F statistic, with an F value greater than 10 indicating sufficient strength to avoid weak bias [23]. Furthermore, we calculated the statistical power of our analysis following the methodology of Brion et al. [39].

### 4.2. MR Analysis

MR analyses were performed using the 2SMR approach with the R package “TwoSampleMR” [34], and a significant association was determined at a *p* < 0.05. The IVW, the maximum likelihood, the MR-Egger and the MR-PRESSO methods were selected for the analysis.

The IVW method combines the effects of all the IVs and, by assuming the validity or invalidity of all the SNPs, sets the global pleiotropy to zero [34]. The selection between IVW FE or random effects was determined by the *p*-value of the heterogeneity test, where a *p* > 0.05 indicated the use of the fixed-effects model. The maximum likelihood approach is based on the direct maximization of the likelihood and provides more accurate confidence intervals than the IVW when there is some uncertainty in the genetic associations [40]. We also employed MR-Egger regression, as it has the capacity to estimate causality using weak or invalid IVs and to provide an estimation of the pleiotropy [41]. MR-PRESSO was implemented due to its capability to detect outliers and to provide an outlier-free estimate [42]. Additionally, weighted median and weighted mode were applied to obtain an unbiased estimate in the presence of some invalid IVs, weighting the contribution of the IVs [43,44]. The median method requires at least half of IVs to be valid, while the mode method assumes the validity of IVs within the larger group of IVs based on their similarity [43,44].

In order to evaluate the effect of each SNP, we carried out a LOO sensitivity analysis, which provides the IVW estimate sequentially excluding one of the IVs at a time [34], applying the tool implemented in the “TwoSampleMR” package (https://elifesciences.org/articles/34408), accessed on 26 September 2023 [34].

## 5. Conclusions

In conclusion, our study points towards a possible causal relationship between increased LTL and a reduced risk of SSc. It is important to interpret these results with caution until further investigation is conducted in this area, acknowledging the inherent limitations of MR studies in fully assessing the assumptions related to the exclusive impact of the IVs on the outcome through the risk factor. Future studies with the aim of clarifying specific associations and evaluating possible mechanisms are warranted.

## Figures and Tables

**Table 1:** Genetic associations of the selected instrumental variables with LTL and SSc.

						LTL		SSc	
SNP	CHR	BP	Gene	Effect Allele	Other Allele	β	*p*	β	*p*
rs11125529	2	54,475,866	*ACYP2*	C	A	−0.056	4.48 × 10^−8^	−0.021	0.478
rs10936599	3	169,492,101	*TERC*	T	C	−0.097	2.54 × 10^−31^	0.051	0.043
rs7675998	4	164,007,820	*NAF1*	A	G	−0.074	4.35 × 10^−16^	−0.004	0.910
rs2736100	5	1,286,516	*TERT*	A	C	−0.078	4.38 × 10^−19^	0.034	0.145
rs9420907	10	105,676,465	*OBFC1*	A	C	−0.069	6.90 × 10^−11^	−0.002	0.974
rs8105767	19	22,215,441	*ZNF208*	A	G	−0.048	1.11 × 10^−9^	0.039	0.297
rs755017	20	62,421,622	*RTEL1*	A	G	−0.062	6.71 × 10^−9^	0.025	0.450

BP: base pair position; CHR: chromosome; LTL: leukocyte telomere length; SSc: systemic sclerosis; β: size effect of the association.

**Table 2:** Association between genetically predicted LTL and risk of SSc.

MR Approach	nSNP	OR (95% CI)	*p*	*p* for Heterogeneity	*p* for Pleiotropy
Inverse-variance weighted FE	7	0.716 (0.528–0.970)	0.031	0.708	NA
Maximum likelihood	7	0.714 (0.525–0.970)	0.031	NA	NA
MR Egger	7	0.397 (0.094–1.670)	0.263	0.686	0.448
Weighted median	7	0.642 (0.438–0.941)	0.023	NA	NA
Weighted mode	7	0.625 (0.411–0.952)	0.071	NA	NA
MR-PRESSO	7	0.716 (0.563–0.911)	0.035	NA	NA

CI: confidence interval; FE: fixed effects; LTL: leukocyte telomere length; MR: mendelian randomization; NA: not applicable; nSNP: number of single-nucleotide polymorphisms in the analysis; OR: odds ratio; PRESSO: pleiotropy residual sum and outlier; SSc: systemic sclerosis.

**Table 3:** Association between genetically predicted LTL and risk of SSc main clinical subtypes.

		lcSSc				dcSSc			
MR Approach	nSNP	OR (95% CI)	*p*	*p* for Heterogeneity	*p* for Pleiotropy	OR (95% CI)	*p*	*p* for Heterogeneity	*p* for Pleiotropy
Inverse-variance weighted FE	7	0.669 (0.468–0.956)	0.027	0.772	NA	0.771 (0.481–1.237)	0.281	0.847	NA
Maximum likelihood	7	0.667 (0.466–0.957)	0.028	NA	NA	0.770 (0.479–1.237)	0.280	NA	NA
MR Egger	7	0.338 (0.062–1.845)	0.266	0.755	0.457	0.389 (0.042–3.646)	0.446	0.804	0.567
Weighted median	7	0.605 (0.393–0.932)	0.023	NA	NA	0.647 (0.360–1.163)	0.146	NA	NA
Weighted mode	7	0.574 (0.345–0.954)	0.076	NA	NA	0.644 (0.329–1.262)	0.247	NA	NA
MR-PRESSO	7	0.669 (0.514–0.872)	0.025	NA	NA	0.771 (0.562–1.058)	0.159	NA	NA

CI: confidence interval; dcSSc: diffuse cutaneous systemic sclerosis; FE: fixed effects; lcSSc: limited cutaneous systemic sclerosis; LTL: leukocyte telomere length; MR: mendelian randomization; NA: not applicable; nSNP: number of single-nucleotide polymorphisms in the analysis; OR: odds ratio; PRESSO: pleiotropy residual sum and outlier; SSc: systemic sclerosis.

## Data Availability

Summary statistics of the GWAS in SSc are available through the NHGRI-EBI GWAS Catalog (https://www.ebi.ac.uk/gwas/downloads/summary-statistics), accessed on 22 February 2023. Other data are available on reasonable request.

## Supplementary Materials

The following supporting information can be downloaded at: https://www.mdpi.com/article/10.3390/ijms242115589/s1.

## References

[1] Denton C.P., Khanna D. (2017). Systemic Sclerosis. Lancet.

[2] Allanore Y., Simms R., Distler O., Trojanowska M., Pope J., Denton C.P., Varga J. (2015). Systemic Sclerosis. Nat. Rev. Dis. Primers.

[3] López-Isac E., Acosta-Herrera M., Kerick M., Assassi S., Satpathy A.T., Granja J., Mumbach M.R., Beretta L., Simeón C.P., Carreira P. (2019). GWAS for Systemic Sclerosis Identifies Multiple Risk Loci and Highlights Fibrotic and Vasculopathy Pathways. Nat. Commun..

[4] Terao C., Kawaguchi T., Dieude P., Varga J., Kuwana M., Hudson M., Kawaguchi Y., Matucci-Cerinic M., Ohmura K., Riemekasten G. (2017). Transethnic Meta-Analysis Identifies and as Susceptibility Genes to Systemic Sclerosis. Ann. Rheum. Dis..

[5] Mayes M.D., Bossini-Castillo L., Gorlova O., Martin J.E., Zhou X., Chen W.V., Assassi S., Ying J., Tan F.K., Arnett F.C. (2014). Immunochip Analysis Identifies Multiple Susceptibility Loci for Systemic Sclerosis. Am. J. Hum. Genet..

[6] Villanueva-Martín G., Martín J., Bossini-Castillo L. (2022). Recent Advances in Elucidating the Genetic Basis of Systemic Sclerosis. Curr. Opin. Rheumatol..

[7] Beretta L., Barturen G., Vigone B., Bellocchi C., Hunzelmann N., De Langhe E., Cervera R., Gerosa M., Kovács L., Ortega Castro R. (2020). Genome-Wide Whole Blood Transcriptome Profiling in a Large European Cohort of Systemic Sclerosis Patients. Ann. Rheum. Dis..

[8] Fang D., Chen B., Lescoat A., Khanna D., Mu R. (2022). Immune Cell Dysregulation as a Mediator of Fibrosis in Systemic Sclerosis. Nat. Rev. Rheumatol..

[9] Keret S., Rimar D., Lansiaux P., Feldman E., Lescoat A., Milman N., Farge D., MATHEC Working Group (2023). Differentially Expressed Genes in Systemic Sclerosis: Towards Predictive Medicine with New Molecular Tools for Clinicians. Autoimmun. Rev..

[10] Heba A.-C., Toupance S., Arnone D., Peyrin-Biroulet L., Benetos A., Ndiaye N.C. (2021). Telomeres: New Players in Immune-Mediated Inflammatory Diseases?. J. Autoimmun..

[11] Shi B., Tsou P.-S., Ma F., Mariani M.P., Mattichak M.N., LeBrasseur N.K., Chini E.N., Lafyatis R., Khanna D., Whitfield M.L. (2023). Senescent Cells Accumulate in Systemic Sclerosis Skin. J. Investig. Dermatol..

[12] Jung S.M., Park K.-S., Kim K.-J. (2022). Integrative Analysis of Lung Molecular Signatures Reveals Key Drivers of Systemic Sclerosis-Associated Interstitial Lung Disease. Ann. Rheum. Dis..

[13] Zhang J., Rane G., Dai X., Shanmugam M.K., Arfuso F., Samy R.P., Lai M.K.P., Kappei D., Kumar A.P., Sethi G. (2016). Ageing and the Telomere Connection: An Intimate Relationship with Inflammation. Ageing Res. Rev..

[14] Rossiello F., Jurk D., Passos J.F., d’Adda di Fagagna F. (2022). Telomere Dysfunction in Ageing and Age-Related Diseases. Nat. Cell Biol..

[15] Usategui A., Municio C., Arias-Salgado E.G., Martín M., Fernández-Varas B., Del Rey M.J., Carreira P., González A., Criado G., Perona R. (2022). Evidence of Telomere Attrition and a Potential Role for DNA Damage in Systemic Sclerosis. Immun. Ageing.

[16] Artlett C.M., Black C.M., Briggs D.C., Stevens C.O., Welsh K.I. (1996). Telomere Reduction in Scleroderma Patients: A Possible Cause for Chromosomal Instability. Br. J. Rheumatol..

[17] Lakota K., Hanumanthu V.S., Agrawal R., Carns M., Armanios M., Varga J. (2019). Short Lymphocyte, but Not Granulocyte, Telomere Length in a Subset of Patients with Systemic Sclerosis. Ann. Rheum. Dis..

[18] Liu S., Chung M.P., Ley B., French S., Elicker B.M., Fiorentino D.F., Chung L.S., Boin F., Wolters P.J. (2021). Peripheral Blood Leucocyte Telomere Length Is Associated with Progression of Interstitial Lung Disease in Systemic Sclerosis. Thorax.

[19] MacIntyre A., Brouilette S.W., Lamb K., Radhakrishnan K., McGlynn L., Chee M.M., Parkinson E.K., Freeman D., Madhok R., Shiels P.G. (2008). Association of Increased Telomere Lengths in Limited Scleroderma, with a Lack of Age-Related Telomere Erosion. Ann. Rheum. Dis..

[20] Boehm F.J., Zhou X. (2022). Statistical Methods for Mendelian Randomization in Genome-Wide Association Studies: A Review. Comput. Struct. Biotechnol. J..

[21] Chen C., Wang P., Zhang R.-D., Fang Y., Jiang L.-Q., Fang X., Zhao Y., Wang D.-G., Ni J., Pan H.-F. (2022). Mendelian randomization as a tool to gain insights into the mosaic causes of autoimmune diseases. Autoimmun. Rev..

[22] Codd V., Nelson C.P., Albrecht E., Mangino M., Deelen J., Buxton J.L., Hottenga J.J., Fischer K., Esko T., Surakka I. (2013). Identification of Seven Loci Affecting Mean Telomere Length and Their Association with Disease. Nat. Genet..

[23] Burgess S., Butterworth A., Thompson S.G. (2013). Mendelian Randomization Analysis with Multiple Genetic Variants Using Summarized Data. Genet. Epidemiol..

[24] Liu M., Luo P., Liu L., Wei X., Bai X., Li J., Wu L., Luo M. (2023). Immune-Mediated Inflammatory Diseases and Leukocyte Telomere Length: A Mendelian Randomization Study. Front. Genet..

[25] Zeng Z., Zhang W., Qian Y., Huang H., Wu D.J.H., He Z., Ye D., Mao Y., Wen C. (2020). Association of Telomere Length with Risk of Rheumatoid Arthritis: A Meta-Analysis and Mendelian Randomization. Rheumatology.

[26] Wang X.-F., Xu W.-J., Wang F.-F., Leng R., Yang X.-K., Ling H.-Z., Fan Y.-G., Tao J.-H., Shuai Z.-W., Zhang L. (2022). Telomere Length and Development of Systemic Lupus Erythematosus: A Mendelian Randomization Study. Arthritis Rheumatol..

[27] Fingerlin T.E., Murphy E., Zhang W., Peljto A.L., Brown K.K., Steele M.P., Loyd J.E., Cosgrove G.P., Lynch D., Groshong S. (2013). Genome-Wide Association Study Identifies Multiple Susceptibility Loci for Pulmonary Fibrosis. Nat. Genet..

[28] Alder J.K., Armanios M. (2022). Telomere-Mediated Lung Disease. Physiol. Rev..

[29] Li G., Larregina A.T., Domsic R.T., Stolz D.B., Medsger T.A., Lafyatis R., Fuschiotti P. (2017). Skin-Resident Effector Memory CD8^+^CD28^−^ T Cells Exhibit a Profibrotic Phenotype in Patients with Systemic Sclerosis. J. Investig. Dermatol..

[30] O’Donovan A., Pantell M.S., Puterman E., Dhabhar F.S., Blackburn E.H., Yaffe K., Cawthon R.M., Opresko P.L., Hsueh W.-C., Satterfield S. (2011). Cumulative Inflammatory Load Is Associated with Short Leukocyte Telomere Length in the Health, Aging and Body Composition Study. PLoS ONE.

[31] Rodier F., Coppé J.-P., Patil C.K., Hoeijmakers W.A.M., Muñoz D.P., Raza S.R., Freund A., Campeau E., Davalos A.R., Campisi J. (2009). Persistent DNA Damage Signalling Triggers Senescence-Associated Inflammatory Cytokine Secretion. Nat. Cell Biol..

[32] Wang Z., Lieberman P.M. (2016). The Crosstalk of Telomere Dysfunction and Inflammation through Cell-Free TERRA Containing Exosomes. RNA Biol..

[33] Adler B.L., Boin F., Wolters P.J., Bingham C.O., Shah A.A., Greider C., Casciola-Rosen L., Rosen A. (2021). Autoantibodies Targeting Telomere-Associated Proteins in Systemic Sclerosis. Ann. Rheum. Dis..

[34] Hemani G., Zheng J., Elsworth B., Wade K.H., Haberland V., Baird D., Laurin C., Burgess S., Bowden J., Langdon R. (2018). The MR-Base Platform Supports Systematic Causal Inference across the Human Phenome. Elife.

[35] Staley J.R., Blackshaw J., Kamat M.A., Ellis S., Surendran P., Sun B.B., Paul D.S., Freitag D., Burgess S., Danesh J. (2016). PhenoScanner: A Database of Human Genotype-Phenotype Associations. Bioinformatics.

[36] Kamat M.A., Blackshaw J.A., Young R., Surendran P., Burgess S., Danesh J., Butterworth A.S., Staley J.R. (2019). PhenoScanner V2: An Expanded Tool for Searching Human Genotype-Phenotype Associations. Bioinformatics.

[37] Machiela M.J., Chanock S.J. (2015). LDlink: A Web-Based Application for Exploring Population-Specific Haplotype Structure and Linking Correlated Alleles of Possible Functional Variants. Bioinformatics.

[38] Lin S.-H., Brown D.W., Machiela M.J. (2020). LDtrait: An Online Tool for Identifying Published Phenotype Associations in Linkage Disequilibrium. Cancer Res..

[39] Brion M.-J.A., Shakhbazov K., Visscher P.M. (2013). Calculating Statistical Power in Mendelian Randomization Studies. Int. J. Epidemiol..

[40] Burgess S., Scott R.A., Timpson N.J., Davey Smith G., Thompson S.G., EPIC-InterAct Consortium (2015). Using Published Data in Mendelian Randomization: A Blueprint for Efficient Identification of Causal Risk Factors. Eur. J. Epidemiol..

[41] Burgess S., Thompson S.G. (2017). Interpreting Findings from Mendelian Randomization Using the MR-Egger Method. Eur. J. Epidemiol..

[42] Verbanck M., Chen C.-Y., Neale B., Do R. (2018). Detection of Widespread Horizontal Pleiotropy in Causal Relationships Inferred from Mendelian Randomization between Complex Traits and Diseases. Nat. Genet..

[43] Bowden J., Davey Smith G., Haycock P.C., Burgess S. (2016). Consistent Estimation in Mendelian Randomization with Some Invalid Instruments Using a Weighted Median Estimator. Genet. Epidemiol..

[44] Hartwig F.P., Davey Smith G., Bowden J. (2017). Robust Inference in Summary Data Mendelian Randomization via the Zero Modal Pleiotropy Assumption. Int. J. Epidemiol..

